# From Machine Learning-Enhanced Proteomics to a Validated Diagnostic Model: A Pipeline for Breast Cancer Biomarker Discovery via Independent and Transcriptomic Corroboration

**DOI:** 10.3390/bioengineering13091040

**Published:** 2026-09-07

**Authors:** Xiaoyan Zhou, Yue Li, Ting Ding, Jiali Liu, Dongdong Tong, Yudong Mu, Nan Xu, Sipeng Li, Hao Meng, Ning Gao, Qian He

**Affiliations:** 1 Department of Clinical Laboratory, Second Affiliated Hospital of Xi’an Jiaotong University, Xi’an 710004, China; xiaoyanzhou10@163.com (X.Z.); liyuelywwcx@126.com (Y.L.); dingting1994@163.com (T.D.); liujiali_911715@163.com (J.L.); nanxu10@stu.xjtu.edu.cn (N.X.); lsp_252@163.com (S.L.); mh-8725@163.com (H.M.); gaoningzq@163.com (N.G.); 2 Department of Cell Biology and Genetics, School of Basic Medical Sciences, Xi’an Jiaotong University Health Science Center, Xi’an 710119, China; tongdd@xjtu.edu.cn; 3 Department of Clinical Laboratory, Shaanxi Provincial Cancer Hospital, Xi’an 710061, China; zt951029@sina.com

**Keywords:** artificial intelligence, proteomics, machine learning, breast cancer, early diagnosis

## Abstract

Early diagnosis of breast cancer (BC) remains challenging. The limited sensitivity and specificity of existing serum tumor markers for reliable clinical application highlight the need to develop a more accurate and efficient screening workflow. This study analyzed serum samples from 255 breast cancer patients and 300 healthy controls using matrix-assisted laser desorption/ionization time-of-flight (MALDI-TOF) mass spectrometry, identifying 58 differentially expressed peptides (37 upregulated, 21 downregulated). Combined with machine learning, peptide identification, and external validation, a complete standardized workflow was established. Nine machine learning (ML) algorithms were employed and compared, including SVM, LightGBM, XGBoost, etc. The models were interpreted using SHAP and LIME to identify key features. Peptides of interest were sequenced via mass spectrometry. Their expression and potential prognostic value were further validated in breast cancer transcriptomic datasets. Nine machine learning algorithms showed favorable discriminatory ability in the study cohort. The LightGBM model achieved an AUC of 0.97 internally and maintained an AUC of 0.88, an accuracy of 0.8543, and a precision of 0.9799 externally. However, after correcting for the markedly elevated prevalence (80.3%) in the external cohort, the positive predictive value (PPV) decreased substantially under real-world screening scenarios, warranting prospective validation in true screening populations. Model interpretation and subsequent sequencing identified six core biomarker peptides: Apolipoprotein A-IV (APOA4), Serum Deprivation Response Protein (SDPR), Alpha-1-Antitrypsin (SERPINA1), Ezrin (EZR), Serglycin (SRGN), and Fibrinogen Alpha Chain (FGA). Transcriptomic corroboration suggested that these molecules were significantly dysregulated in breast cancer tissues and showed univariate prognostic associations with patient survival. These findings demonstrated the potential of a proteomics-driven integrated machine learning pipeline as a proof-of-concept auxiliary risk-stratification tool for enhancing early breast cancer diagnosis, warranting further prospective validation in real-world screening cohorts before clinical translation.

## 1. Introduction

Breast cancer (BC) is the most prevalent malignant tumor among women worldwide, with an estimated 2.3 million (ranking first among all cancers) new cases and 666,000 deaths (ranking second) reported in 2022 [1]. In China, 357,000 new breast cancer cases were documented in 2022, ranking sixth among female malignancies, and the number of deaths reached 109,000, making it the fourth leading cause of cancer-related deaths in women [2]. The high disease burden persists, especially in regions with inadequate access to standardized screening programs [3]. Early diagnosis is pivotal for improving cure rates and patient survival, as the 5-year survival rate of early breast cancer (stage I–II) exceeds 90%, while that of advanced-stage disease (stage III–IV) drops to less than 30% [3,4,5,6].

Current clinical diagnostic methods for breast cancer include mammography [7], ultrasound [8], Magnetic Resonance Imaging (MRI) [9,10], core needle biopsy [11], and tumor biomarker detection [12]. Although these methods have certain clinical value, they face inherent limitations: mammography has suboptimal sensitivity in young women with dense breast tissue [3]; ultrasound is highly operator-dependent, with inter-observer agreement particularly poor for early lesions [6]; MRI is costly and unsuitable for large-scale screening [10,13]; invasive biopsies may cause bleeding, infection, or breast tissue scarring [14]; and single tumor biomarkers such as carcinoembryonic antigen (CEA) and carbohydrate antigen 15-3 (CA15-3) have a specificity of less than 70% for early diagnosis, leading to high false-positive rates [12]. However, clinical translation of proteomics-derived biomarkers remains challenging: single biomarkers often exhibit low specificity due to cross-reactivity with non-cancerous conditions (e.g., CA15-3 elevation in benign breast disease); combining multiple biomarkers improves efficacy but still fails to meet early screening requirements [15,16]; and antibody-dependent diagnostic kits face obstacles such as difficult antibody preparation, poor stability, and high development costs. Thus, developing novel, non-invasive, and high-precision diagnostic technologies is urgently needed.

Proteomics has become a key strategy for discovering tumor biomarkers by identifying specific protein/peptide signatures in body fluids such as serum or plasma [17,18]. Among the emerging Mass Spectrometry (MS) technologies, Data-independent acquisition (DIA) has become a transformative approach in cancer proteomic research [19]. Unlike conventional Data-dependent Acquisition (DDA), DIA systematically acquires fragment-ion spectra for all precursor ions within a predefined mass-to-charge (*m*/*z*) range, enabling unbiased and comprehensive proteome profiling [20]. This strategy markedly reduces missing-value issues and offers substantial improvements in quantitative accuracy, precision, and reproducibility over traditional methods [20]. DIA-MS has demonstrated remarkable potential in tumor molecular subtyping (e.g., colorectal, breast, and prostate cancers), biomarker discovery, therapeutic target identification, and monitoring of treatment response [20,21]. Notably, DIA is compatible with clinically available specimens, including formalin-fixed paraffin-embedded (FFPE) tissues, and has been successfully integrated into large-scale proteomic initiatives such as the Clinical Proteomic Tumor Analysis Consortium (CPTAC) [21]. Nevertheless, the clinical translation of DIA-MS still faces challenges, including the complexity of data interpretation and the lack of standardized protocols across different platforms [20,22].

Following the discovery phase, targeted proteomics techniques are indispensable for the validation of candidate biomarkers. Multiple Reaction Monitoring (MRM) and Parallel Reaction Monitoring (PRM) are the two most widely used targeted quantitation methods [20,23]. PRM, an advanced evolution of MRM, leverages high-resolution, high-accuracy mass spectrometers (e.g., Orbitrap instruments) to combine the high selectivity of quadrupole mass filtering with the high resolving power and mass accuracy of Orbitrap analyzers, allowing selective detection and relative/absolute quantitation of target proteins and peptides [20]. PRM exhibits high selectivity, excellent reproducibility, and high throughput for the detection of low-abundance proteins and has been extensively applied in early cancer detection studies. By monitoring tumor-specific metabolite or protein marker levels, PRM provides robust support for the development of novel diagnostic tools [20,24].

In parallel with MS-based strategies, large-scale affinity-based proteomic platforms, represented by Olink and SomaScan, have emerged as powerful tools in clinical proteomics [20,25]. These platforms are distinguished by their high throughput, superior sensitivity, and broad dynamic range [26]. The Olink technology is based on the Proximity Extension Assay (PEA), which employs pairs of antibodies to recognize target proteins and uses DNA-labeled probes for quantitation, enabling simultaneous measurement of 92 to 3072 proteins in a single experiment [25]. The SomaScan platform utilizes Slow Off-rate Modified Aptamers (SOMAmer) and its latest version (v5.0) can quantify more than 7500 proteins simultaneously in just 55 µL of plasma or serum [20,23]. Recent studies have systematically compared the performance of the SomaScan, Olink, and MS platforms in plasma proteomic analyses, covering over 13,000 proteins, and have revealed their complementary advantages in detection coverage, sensitivity, and precision [22,27]. In oncology, Olink and SomaScan have been widely applied to decipher the tumor microenvironment and to discover diagnostic/prognostic biomarkers in blood and ascites, facilitating clinical translation [20].

In summary, the three technology families—DIA-MS for unbiased discovery, PRM/MRM for targeted verification, and Olink/SomaScan for high-plex affinity-based detection—each have distinct strengths and are mutually complementary, together forming the current technical matrix for cancer diagnostic proteomics. However, irrespective of the technological path, the translation from biomarker discovery to routine clinical application continues to face persistent challenges, including standardization of pre-analytical procedures, sensitivity to low-abundance proteins, and cross-platform data comparability [22,27].

Despite the remarkable capabilities of these advanced proteomic platforms, their translation into routine cancer diagnostics remains hindered by several persistent technical and translational challenges. First, the detection of low-abundance proteins—which often constitute the most promising diagnostic biomarkers—is severely constrained by the immense dynamic range of the human plasma proteome, where protein concentrations span 10 orders of magnitude [28,29]. Highly abundant proteins such as albumin and immunoglobulins, which account for more than 90% of the total protein mass, can mask signals from clinically relevant low-concentration analytes [30]. State-of-the-art mass spectrometers typically provide an intra-scan dynamic range of approximately 10^3^, generally insufficient for direct low-level biomarker detection from human plasma [31]. This limited dynamic range necessitates extensive fractionation or depletion steps, which in turn introduce additional variability and reduce throughput [31]. Second, despite recent improvements, throughput remains a major bottleneck for clinical implementation: MS-based methods, while highly informative, require prolonged acquisition times and complex data processing [32], whereas affinity-based platforms, though faster, still face scalability and cost constraints in high-volume clinical settings [33]. Third, pre-analytical variability—including differences in sample collection, processing, storage, and freeze–thaw cycles—constitutes a critical source of irreproducibility [34]. Studies have shown that high-speed centrifugation can reduce detectable protein numbers to only 30% of the original plasma, and long-term freezer storage gradually and significantly diminishes proteome coverage [34], substantially affecting quantitative accuracy and inter-laboratory comparability [35]. Finally, and perhaps most importantly, there exists a substantial translational gap from biomarker discovery to commercially available clinical kits: the vast majority of candidate protein signatures identified in discovery-phase studies fail to survive rigorous clinical validation. Indeed, comprehensive discovery-based proteomics faces validation failure rates exceeding 90% [30]. Despite over 40,000 scientific manuscripts published in clinical proteomics to date, only a handful of examples of actual clinical application can be found, and none are in wide use [36]. The reasons for this translational gap are multifaceted—poor analytical specificity, lack of standardized reference materials [35], underpowered studies lacking reproducibility [37], and failure to meet regulatory requirements for clinical diagnostic devices all contribute to this persistent discovery-to-clinic divide. Collectively, these multifaceted challenges underscore the urgent need for innovative strategies that can enhance sensitivity, streamline workflows, minimize pre-analytical bias, and bridge the discovery-to-clinic divide.

The rapid advancement of Artificial Intelligence (AI) has revolutionized cancer diagnosis by enabling the analysis of high-dimensional omics data [21,22]. Machine Learning (ML) algorithms can identify complex patterns in genomics, proteomics, and imaging data that are undetectable by traditional statistical methods [23]. ML models analyzing prostate cancer proteomics data achieved an area under the curve (AUC) > 0.87 for malignant phenotype identification [24]. Building on these advancements, we developed an AI-driven pipeline integrating machine learning with mass spectrometry-based proteomics to establish a breast cancer diagnostic model. By analyzing serum data from 255 patients with breast cancer and 300 healthy controls using nine machine-learning algorithms and an independent temporal validation cohort, our multi-dimensional models achieved near-perfect diagnostic accuracy (AUC = 0.97), outperforming single-marker approaches and providing a promising antibody-independent diagnostic alternative.

## 2. Materials and Methods

### 2.1. Proteomics Workflow for Breast Cancer Patients’ Serum

This study employed Matrix-Assisted Laser Desorption/Ionization Time-of-Flight Mass Spectrometer (MALDI-TOF MS, Zybio EXS2600, Chongqing, China)-based serum proteomic profiling. Raw spectral data were processed via curve-fitting and normalization to reduce technical variability (coefficient of variation < 5% for peak intensity). A normalized peak intensity matrix was analyzed using ML classifiers to identify discriminative features. Total Ion Current (TIC) normalization was applied to each spectrum. High-priority peaks (feature importance > 0.05) were annotated for biological validation. All data analyses were performed using Python 3.7.0, with visualization via Matplotlib (version 3.7.0) and Seaborn (version 0.13.2).

Normalization was performed in two steps. First, total ion current (TIC) scaling was applied within a restricted *m*/*z* window (1500–10,000 Da) to exclude matrix and non-peptide noise. Second, median centering was performed on each peak feature (column) by subtracting the median intensity across all samples, which reduces systematic bias and improves model convergence. Differential peaks were identified using a two-sample *t*-test, followed by Benjamini–Hochberg correction for multiple comparisons across all identified peaks. Peaks with an adjusted *p*-value < 0.001 and |log2 fold change| > 1 were considered differential.

### 2.2. Study Cohort and Sample Preparation

A prospective case–control study was conducted at the Second Affiliated Hospital of Xi’an Jiaotong University; a total of 555 participants were enrolled, comprising 255 treatment-naïve BC patients and 300 age-matched healthy controls. All breast cancer patients were recruited and diagnosed at the Second Affiliated Hospital of Xi’an Jiaotong University (September–December 2025). Patients with other malignancies, major systemic diseases, other diseases affecting kidney function, or prior anticancer therapies were excluded. Healthy controls were recruited from individuals undergoing routine physical examinations at the same center during a comparable period. To enable a robust assessment of model generalizability, the independent temporal validation cohort was recruited after the development cohort, using identical eligibility criteria. The study was conducted in accordance with the Declaration of Helsinki and was approved by the Institutional Ethics Committee (No. 2025–212) and conducted in accordance with the Declaration of Helsinki, with broad informed consent (as the samples were derived from the residual serum after testing and did not involve patients’ personal information or repeated blood draws). Blood serum samples (3 mL) were collected into anticoagulant-free vacuum tubes and centrifuged (3500 rpm, 10 min, 4 °C, Michael, Changsha, China). Duplicates of 50 µL serum samples were stored at −80 °C (to avoid repeated freezing and thawing) and analyzed within 2 h after thawing to prevent protein degradation. Mass spectrometric profiling was conducted in strict adherence to standardized operating procedures throughout sample handling, instrument operation, and data acquisition to ensure reproducibility and minimize batch effects. For the complete workflow, please refer to Supplementary Figure S1.

### 2.3. MALDI-TOF MS Analysis and Data Preprocessing

Serum peptides were enriched using an immobilized metal affinity-magnetic bead protocol to deplete high-abundance proteins. Following binding and washing, the eluted peptides were mixed with α-cyano-4-hydroxycinnamic acid matrix and analyzed in triplicate via MALDI-TOF mass spectrometry in positive linear mode (mass range: 1–15 kDa). Raw spectra were processed using a custom Python workflow. This included smoothing, baseline correction, noise reduction, and automated peak detection. Peaks were aligned across samples to correct for mass drift and missing values were imputed as zero or linearly interpolated from adjacent *m*/*z* bins for downstream analysis.

Samples were processed and acquired in randomized order, with case and control samples interleaved throughout all experimental batches; the operator was blinded to clinical status. Internal quality control and batch-to-batch consistency were ensured as follows: (1) QC samples were included in every batch and processed alongside study samples, with intra-batch deviation maintained below 5%; (2) Bridging QC samples were inserted between adjacent batches to verify inter-batch reproducibility; and (3) Weekly external calibration was followed by three consecutive QC measurements from MALDI spots, requiring CVs of *m*/*z* and peak intensity for characteristic peaks to be <3%. Normalization was performed in two steps. First, total ion current (TIC) scaling was applied within a restricted *m*/*z* window (1500–10,000 Da) to exclude matrix and non-peptide noise. Second, median centering was performed on each peak feature by subtracting the median intensity across all samples, which reduces systematic bias and improves model convergence.

### 2.4. Machine Learning Algorithms-Enhanced Prediction for Breast Cancer

The dataset was split into a training cohort (70%) and an independent test set (30%) via stratified sampling to preserve class distribution (breast cancer:control = 1:1.18). Dataset splitting was conducted upfront, preceding any feature selection or model building. The independent test set was strictly sequestered from the entire development cycle and was accessed only at the final stage to gauge the model’s generalizability; it played no role in feature filtering, classifier training, or hyperparameter tuning. Nine ML classifiers were employed, covering traditional and ensemble algorithms. Traditional algorithms include Logistic Regression, *K*-nearest neighbors (KNN, *k* = 5), Support Vector Machine (SVM, linear kernel), Gaussian Naive Bayes (GaussianNB) and Decision Tree (max depth = 10); Ensemble algorithms involve Random Forest (100 trees), Extreme Gradient Boosting (XGBoost, learning rate = 0.1), Adaptive Boosting (AdaBoost, 50 estimators) and light gradient boosting machine (LightGBM, 100 trees, v3.3.2). The primary metrics were AUC-ROC, accuracy (proportion of correct predictions), precision (proportion of true positives among predicted positives), recall (proportion of true positives identified) and F1 score (harmonic mean of precision and recall).

To determine whether the comparison among algorithms was sensitive to the hyperparameter-tuning strategy, we additionally performed a uniform nested cross-validation analysis. Five-fold stratified cross-validation was used for the outer evaluation loop, and four-fold stratified grid search was performed within each outer training fold using ROC-AUC as the common optimization criterion. Median imputation and Z-score standardization were incorporated into the modeling pipeline and fitted exclusively within the corresponding training folds. The same outer-fold partitions and evaluation criteria were used for all nine algorithms, while algorithm-specific parameter grids were retained because different classifiers have different applicable hyperparameters. This analysis was a nested classifier-tuning analysis conditional on the locked feature panel, rather than an unbiased end-to-end validation of the complete discovery workflow, and was treated as a supplementary sensitivity analysis and did not replace the primary train/test evaluation. Detailed search spaces are provided in Supplementary Table S1.

### 2.5. Feature Selection for Interpretative and Clinical Decision-Making Model

Three complementary methods were used to identify biologically relevant peptides, reducing feature redundancy and improving model interpretability. Following the initial model development, we employed a multi-faceted strategy to identify the most informative peptide peaks. First, the feature importance derived from Local Interpretable Model-agnostic Explanations (LIME) and SHapley Additive exPlanations (SHAP) was integrated with the net benefit assessment from Decision Curve Analysis (DCA). Based on a composite ranking from these analyses, the top 10 candidate peptide peaks were selected for downstream validation. The optimality of the final peak signature was assessed by leave-one-out (LOO) analysis (sequentially omitting each peak) and forward stepwise addition (sequentially adding the remaining candidate peaks), with model performance compared using the area under the ROC curve (AUC). The clinical utility of each peak was assessed via DCA to estimate its net benefit across a range of clinically reasonable threshold probabilities. This two-stage process, initial multi-criteria screening followed by individual performance validation, enabled us to further refine the list and prioritize the peptide peaks with the highest combined contribution to model interpretability and robust diagnostic power.

### 2.6. Biomarker Annotation and Model Optimization

To experimentally verify the model predictions, the top 10 scoring candidate peptides were identified via LC-ESI-MS/MS for sequence-level confirmation. Following this validation, the predictive performance for this refined set of peptides was re-evaluated. This involved re-computing the AUC, confusion matrix and other key performance metrics under the identical training and testing protocol used in the initial model assessment.

### 2.7. Internal Verification and Independent Temporal Validation

Internal validation in the primary analysis included evaluation on the held-out test subset and stratified five-fold cross-validation. The supplementary nested cross-validation analysis was used to assess the sensitivity of model performance to hyperparameter selection and preprocessing. For independent temporal validation, the locked model was assessed using a prospectively collected independent cohort comprising 100 healthy controls and 408 patients with histologically confirmed breast cancer. No external-cohort samples were used for model training or hyperparameter selection. The same inclusion/exclusion criteria and ethical approvals (including informed consent) used for the training cohort were also applied to the external validation set.

### 2.8. Bioinformatics Analysis and Transcriptomic Corroboration

To provide transcriptomic corroboration and biological context interpretation for the genes encoding the serum peptide peaks of interest (with the explicit clarification that this analysis is not intended as a direct cross-omics validation of the serum peptide abundance, sequence, or diagnostic properties), we integrated transcriptomic data from breast cancer tissues in The Cancer Genome Atlas Breast Invasive Carcinoma (TCGA-BRCA) and matched normal tissue data from Genotype-Tissue Expression (GTEx). Expression values underwent log2(x + 0.001) transformation. Differential expression between breast tumor and normal samples was assessed using the unpaired Wilcoxon rank-sum test in *R* (v3.6.4). For prognostic value survival analysis, samples with follow-up times shorter than 30 days in the TCGA-BRCA were excluded. The association between gene expression levels and overall survival in breast cancer patients was evaluated by fitting Cox proportional hazards regression models with the survival *R* package (v3.2-7), and statistical significance was assessed via the log-rank test. Functional enrichment analysis of differentially expressed genes was conducted using ClusterProfiler (v4.12.6) for Gene Ontology (GO) and Kyoto Encyclopedia of Genes and Genomes (KEGG) pathways. Terms with a *p*-value < 0.05 and a False Discovery Rate (FDR) < 0.1 were considered significantly enriched. It should be emphasized that tissue-derived mRNA expression reflects transcriptional regulation, which is biologically distinct from the abundance, proteolytic origin, and activity of serum peptides. Consequently, these results serve only to explore the potential functional relevance and univariate prognostic trends of the encoding genes in tumor tissues and do not constitute validation of the serum peptide biomarkers.

## 3. Results

### 3.1. General Characteristics of Study Participants

This study included only female participants. The median (IQR) age was 55 (46–64) years in the breast cancer group and 53 (42–67) years in the healthy control group. The Kruskal–Wallis test confirmed no statistically significant difference in age distribution between the groups, supporting the internal validity of the comparative analyses. The independent temporal validation cohort had a comparable age distribution to that of the development cohort (Supplementary Table S2).

### 3.2. Differential Peptide Screening

**Figure 1:**
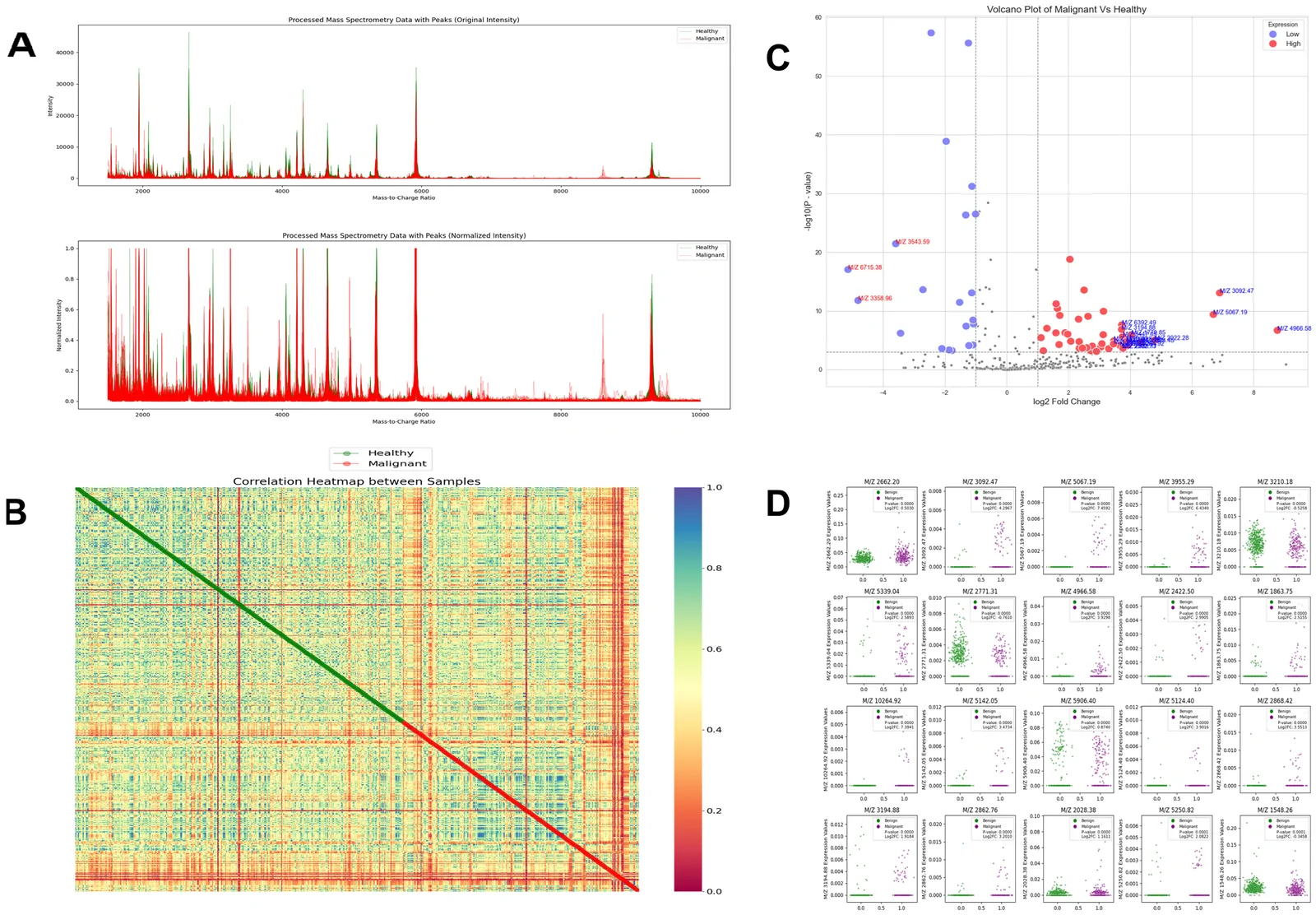
Processing of mass spectrometry data and identification of differentially expressed peptides. (**A**) Representative overlay of raw spectral peaks (**top**) and normalized profiles from all samples (**bottom**). The cohort included 255 breast cancer patients and 300 healthy controls. (**B**) Inter-sample correlation matrix, with healthy controls in green and breast cancer samples in red. (**C**) Volcano plot highlighting peptides significantly upregulated (red) or downregulated (blue) in breast cancer compared to healthy donors. (**D**) List of the 20 most prominent peptides identified during data processing.

MALDI-TOF MS identified polypeptide peaks in the 1000–10,000 Da range, with excellent reproducibility in triplicate measurements (Pearson correlation coefficient > 0.95). The mass spectrometry peaks from the original time-of-flight mass spectrometry data were overlaid after fitting and normalization (Figure 1A). 3D fitting images revealed that differential peaks were mainly distributed in the 1500–6000 Da range (87.3% of significant peaks). The correlation analysis heatmap showed distinct peptide profiles between groups (Figure 1B). A total of 58 differential peaks were identified using Benjamini–Hochberg-adjusted *p* < 0.001 and |log2 fold change| > 1: 37 upregulated and 21 downregulated in the volcano plot (Figure 1C). The top 20 peptides were shown in Figure 1D.

### 3.3. Machine Learning Model Performance Evaluation

**Figure 2:**
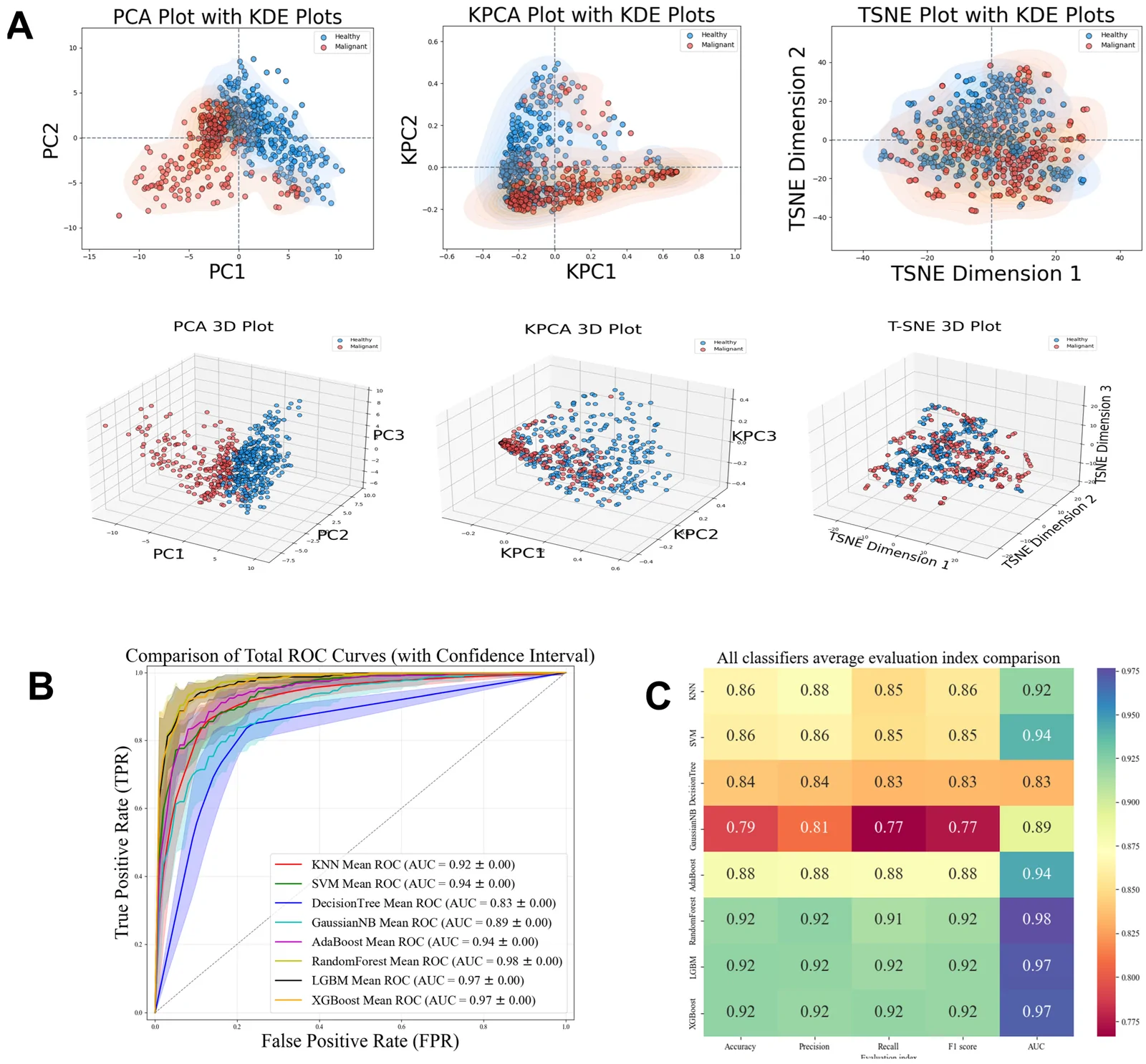
Performance comparison of machine learning models for diagnostic prediction. (**A**) Dimensionality reduction of mass spectrometry data using PCA, KPCA, and *t*-SNE, presented in two- and three-dimensional views. Shaded areas indicate sample cluster distributions. (**B**) Integrated receiver operating characteristic (ROC) curves reflecting the true positive rate (TPR) versus false positive rate (FPR) across models. (**C**) Model evaluation based on accuracy, precision, recall, and F1-score for each machine learning algorithm tested. Panels (**B**,**C**) present the primary held-out test-set results after model selection within the training subset; the uniform nested cross-validation sensitivity analysis is reported separately in Supplementary Tables S3 and S4.

Dimensionality reduction via Principal Component Analysis (PCA), kernel principal component analysis (KPCA), and *t*-distributed stochastic neighbor embedding (*t*-SNE) showed distinct clustering of breast cancer and control samples, with minimal overlap (Figure 2A). All data were split into training and test sets in a 7:3 ratio, followed by analysis of differential peptide peaks using nine ML algorithms. ROC curves and confusion matrices were used to evaluate the performance of each model. The nine machine learning models showed varying but generally favorable diagnostic performance (Figure 2B), with mean AUC values ± standard deviation (SD): for example, SVM: AUC = 0.94 ± 0.01, LightGBM: AUC = 0.97 ± 0.01, XGBoost: AUC = 0.97 ± 0.01. Integration of ROC curves demonstrated strong discriminative ability across all models. The supplementary nested cross-validation analysis yielded a mean ROC-AUC of 0.94 (95% CI: 0.949–0.979) across the outer test folds, which was closely mirrored by the findings from the primary 70%/30% split-sample validation. Key performance metrics, including accuracy, precision, recall, and F1 score, characterized model discrimination and classification performance (Figure 2C).

To assess whether these findings were sensitive to the hyperparameter-selection strategy, we performed an additional nested cross-validation analysis using identical outer folds and a common ROC-AUC optimization criterion for all nine algorithms. The resulting performance pattern was consistent with the primary analysis. XGBoost, Random Forest, LightGBM, and SVM achieved out-of-fold AUCs of 0.974, 0.974, 0.965, and 0.965, respectively. The 95% confidence intervals of the leading models substantially overlapped, indicating broadly comparable discrimination rather than the unique superiority of a single algorithm. Complete results are provided in Supplementary Tables S3 and S4.

Using the out-of-fold LightGBM predictions from the nested cross-validation, calibration and bootstrap-based feature-stability analyses show that it yielded an ROC-AUC of 0.965 and a Brier score of 0.060 (95% CI, 0.043–0.077). The calibration intercept was 0.009 (95% CI, −0.287–0.275), and the calibration slope was 0.435 (95% CI, 0.363–0.550) (Supplementary Figure S2). Overall, the internally validated LightGBM model demonstrated strong discriminative ability and a low Brier score; however, the calibration slope being substantially below unity suggests that the predicted probabilities were more extreme than the observed outcome frequencies, highlighting the need for recalibration in future prospective cohorts.

### 3.4. Feature Selection Results

**Figure 3:**
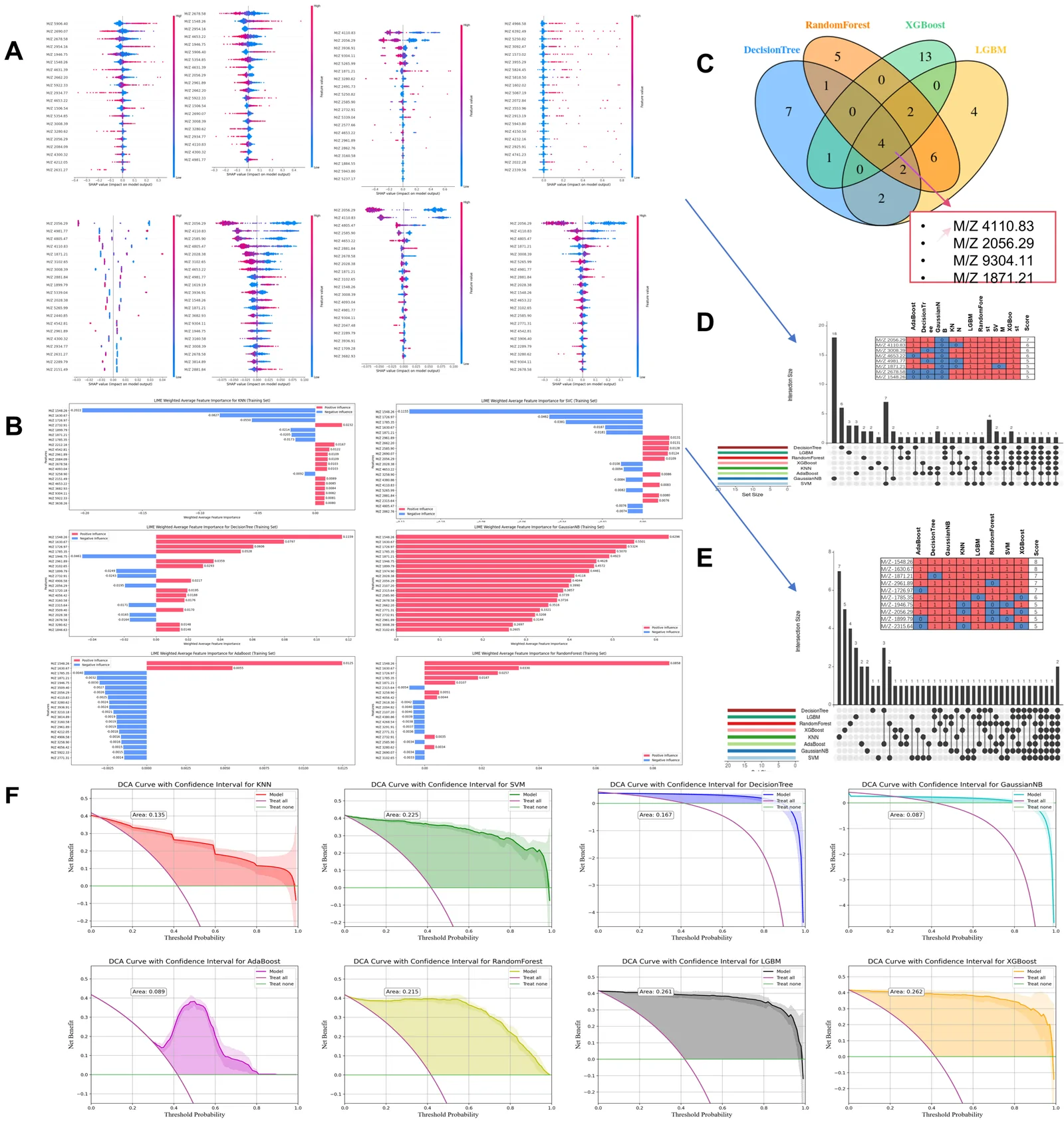
Identification of significant peptide features across machine learning models. (**A**) Key differential peptides ranked by SHAP value importance across various algorithms. (**B**) Prominent differential peptides highlighted through LIME-based interpretation. (**C**) Cross-validated feature importance scores from decision tree, random forest, LightGBM, and XGBoost classifiers. (**D**,**E**) Explanatory visualizations from SHAP (**D**) and LIME (**E**) analyses for each model. (**F**) Decision curve analysis (DCA) evaluating the clinical utility of each algorithm.

Feature importance values from SHAP and LIME identified 20 high-performance differential peptides (Figure 3A,B). Cross-validation of decision tree, random forest, LightGBM, and XGBoost models revealed 4 key differential peaks with consistent importance across algorithms; they are: *m*/*z* 4110.83, *m*/*z* 2056.29, *m*/*z* 9304.11, *m*/*z* 1871.21 (Figure 3C). LIME values provided instance-specific explanations, showing positive contributions from the four key peaks (Figure 3D). SHAP values quantified individual feature contributions, showing that *m*/*z* = 4110.83 had the strongest positive correlation with breast cancer diagnosis (Figure 3E). Integration of the three indicators optimized feature selection, reducing dimensionality while preserving diagnostic efficacy. Neither removal of any single peak nor addition of other candidate proteins substantially altered the AUC, indicating that the ten-peak panel achieved an optimal balance between parsimony and predictive power (Supplementary File S1). Decision curve analysis (DCA) showed that all models had higher net benefits than “treat-none” and “treat-all” strategies across all decision thresholds (0.1–0.9), with LightGBM and XGBoost exhibiting the highest net benefit (0.262 ± 0.03 at threshold = 0.3) (Figure 3F).

### 3.5. Single Peptide Clinical Indicator Analysis

**Figure 4:**
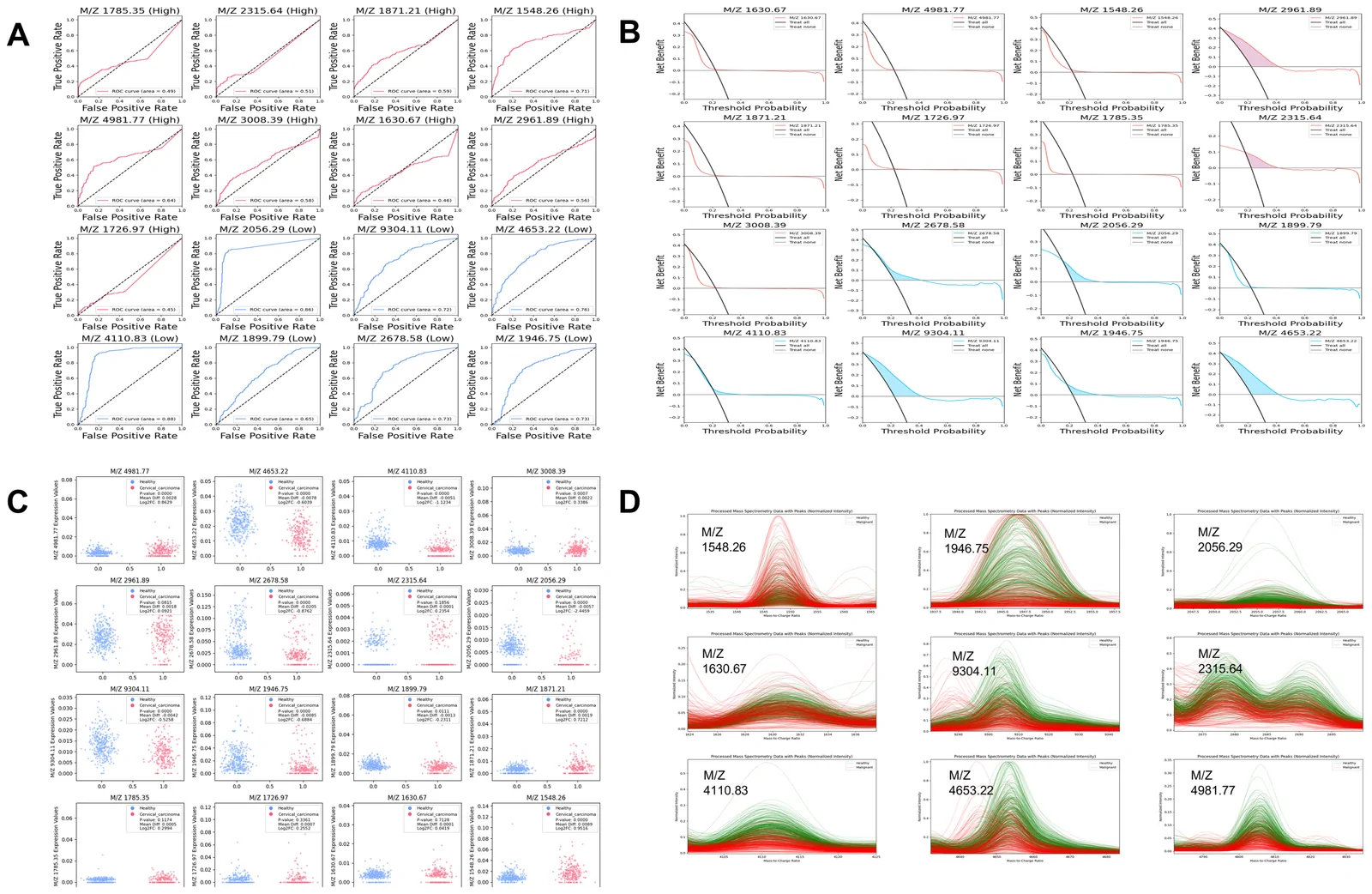
Diagnostic and clinical utility assessment of individual differential peptides. (**A**) ROC curve analysis evaluating the diagnostic performance of individual upregulated (red) and downregulated (blue) peptides. (**B**) Decision curve analysis (DCA) of individual peptides compared to reference lines, with upregulated and downregulated peptides shown in red and blue, respectively. (**C**) Comparison of expression levels for the selected differential peptide between groups. (**D**) Overlay of normalized spectral peaks representing the expression profile of the individual differential peptide.

To assess the clinical potential of the candidate features identified across our comparative models, we first evaluated the standalone diagnostic performance of each feature using receiver operating characteristic (ROC) curve analysis. Individual features demonstrated suboptimal variable discriminatory capacity, with AUC values ranging from 0.45 to 0.88 (Figure 4A). Notably, the feature at *m*/*z* 4110.83 achieved the highest AUC = 0.88, followed by *m*/*z* 2056.29 (AUC = 0.86) and *m*/*z* 4653.22 (AUC = 0.76). Subsequent DCA was conducted to quantify the clinical net benefit of utilizing each feature as a diagnostic marker across a spectrum of threshold probabilities. The DCA curves (Figure 4B) showed that the prediction models based on several features provided a higher net benefit compared to the “treat-all” or “treat-none” strategies within a clinically relevant threshold range. Furthermore, comparative analysis confirmed statistically significant differences in the expression levels of these selected peptides between the experimental and control groups (Figure 4C). The corresponding normalized mass spectrometry peak profiles for these key features are presented in Figure 4D, visually corroborating their differential abundance.

### 3.6. Annotation and Identification for Potential Biomarkers

The top 10 peaks with the highest contribution scores in the model were subjected to LC-ESI-MS/MS sequencing and UniProt annotation. Among these candidate peaks, six were successfully mapped to unique peptide sequences and identified as fragments derived from the following proteins: apolipoprotein A-IV (APOA4), serum deprivation response (SDPR), alpha-1-antitrypsin (SERPINA1), ezrin (EZR), serglycin (SRGN), and fibrinogen alpha chain (FGA). Three peaks were not identified, and *m*/*z* 1630.67, and the peaks at *m*/*z* 1630.67 and *m*/*z* 1871.21 were derived from the same gene (Table 1). For details of peptide identification via LC-ESI-MS/MS sequencing and more physiology of the found protein, please refer to Supplementary File S2 and Supplementary Table S5, respectively.

### 3.7. Diagnostic Efficacy from Internal/External Verification

**Figure 5:**
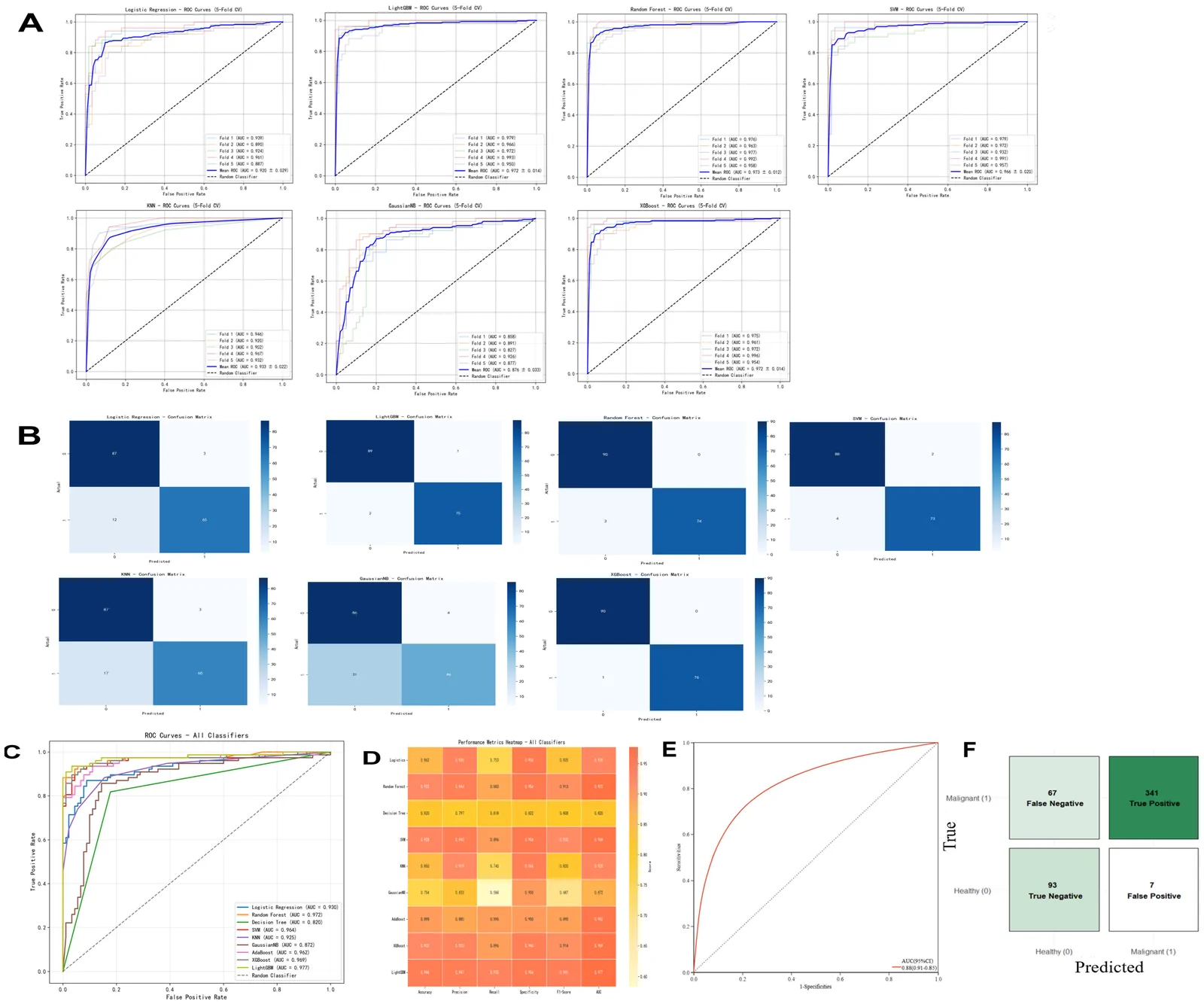
Diagnostic efficacy from internal/external verification. (**A**–**C**) ROC curves, AUC and diagnostic performance values (including accuracy, precision, recall) of the differential molecules under different machine learning algorithms through internal 5-fold stratified cross-validation. (**D**) Confusion matrices illustrating classification outcomes. The matrix displays the correspondence between predicted and actual labels, where diagonal entries (top-left and bottom-right) represent correct classifications (TP and TN), and off-diagonal entries indicate misclassifications (FP and FN). (**E**) ROC curves and AUC values of the LightGBM model through independent external verification (breast cancer: *n* = 408, healthy control: *n* = 100). (**F**) Confusion matrices of the LightGBM model through independent external verification.

Unsupervised clustering (bisecting *K*-means, BIRCH) with internal 5-fold stratified cross-validation showed that the ten peaks effectively distinguish breast cancer from controls. The nine ML models based on these molecules as differential indicators achieved excellent diagnostic performance: SVM: Total mean AUC = 0.966 ± 0.020; Random forest: Total mean AUC = 0.973 ± 0.012; LightGBM: Total mean AUC = 0.972 ± 0.014; KNN: Total mean AUC = 0.973 ± 0.022; XGBoost: Total mean AUC = 0.972 ± 0.014; Gaussian Naive Bayes: Total mean AUC = 0.876 ± 0.033 (Figure 5A–C). The confusion matrix results for each classifier are presented in Figure 5D.

Following the development of our multi-peptide predictive model, we performed an independent temporal validation to rigorously assess its generalizability and clinical translational potential. This validation was conducted on a prospectively collected, held-out cohort comprising breast cancer specimens and matched healthy control samples that were not involved in any prior model training or tuning steps. The model demonstrated robust and consistent diagnostic performance in this independent setting. The receiver operating characteristic (ROC) analysis yielded an area under the curve (AUC) of 0.88 (95% CI: 0.85–0.91) (Figure 5E), indicating a high level of discriminatory accuracy. The corresponding confusion matrix, derived from applying the optimal probability threshold determined during development, is presented in Figure 5F. Overall, the model achieved an accuracy of 85.43% on the external validation set, effectively distinguishing between breast cancer patients and healthy individuals. These external validation results confirm that the identified peptide signature maintains strong predictive power beyond the initial discovery cohort. The AUC of 0.88, coupled with an accuracy exceeding 85%, underscores the model’s reliability and potential utility as a non-invasive diagnostic tool, warranting further investigation in larger, multi-center populations.

### 3.8. Bioinformatics Analysis Results

**Figure 6:**
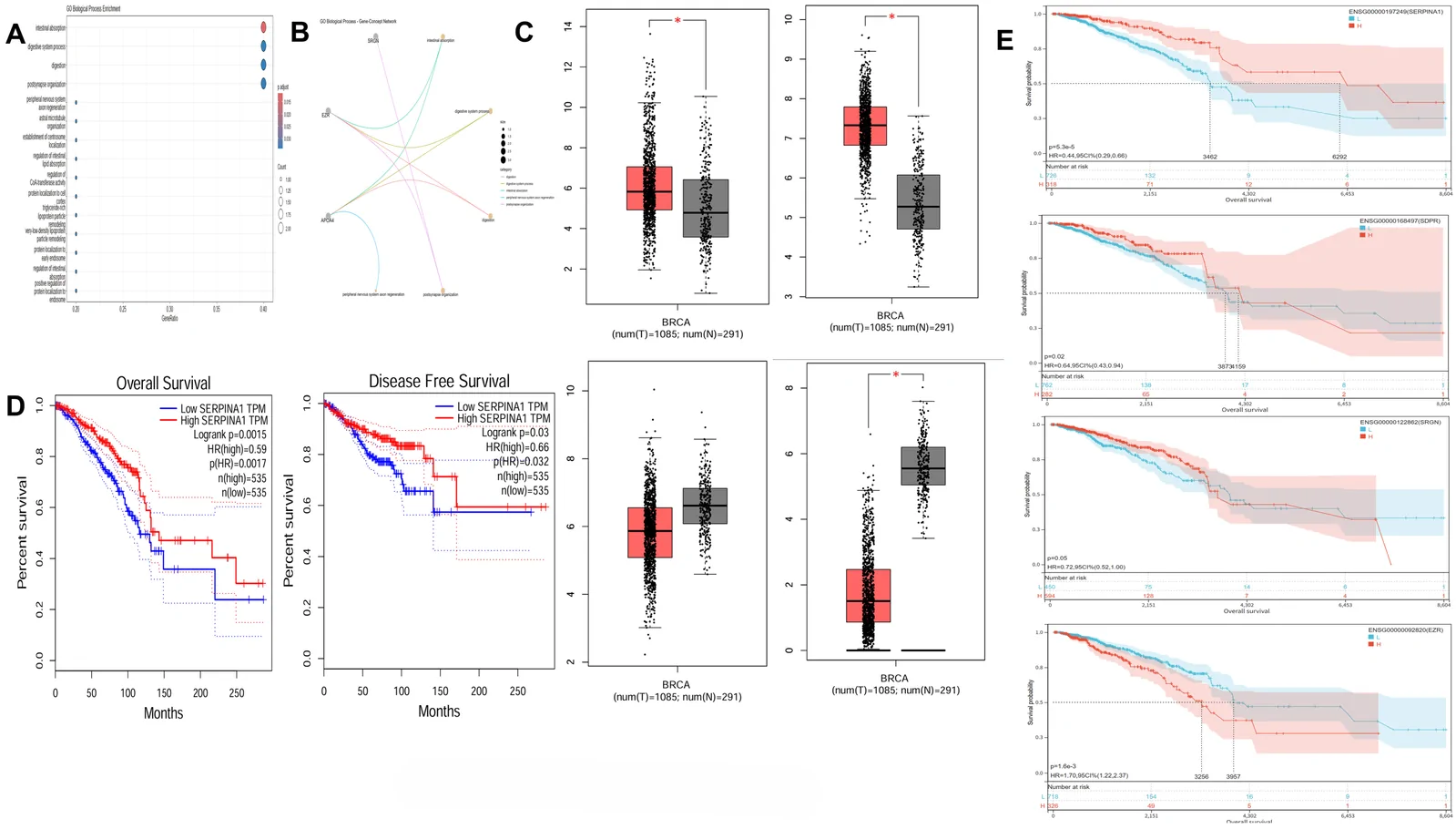
Transcriptomics and bioinformatics analysis of six core biomarkers in breast cancer. (**A**,**B**) GO and KEGG pathway enrichment analysis of the six candidate genes. (**C**) Differential expression analysis of *SERPINA1, EZR, SRGN*, and SDPR between breast cancer tissues (from TCGA-BRCA) and normal breast tissues (from GTEx). (**D**) Kaplan–Meier(K-M) survival curves depicting the association between *SERPINA1* expression and patient outcomes in breast cancer. (**Left**) Overall Survival (OS), log-rank *p* = 0.0015. (**Right**) Disease-Free Survival (DFS), log-rank *p* = 0.03. (**E**) Kaplan–Meier survival analyses summarized the potential prognostic impact of the candidate genes (*EZR, SRGN, SDPR*) on Overall Survival in breast cancer, presented as Hazard Ratios (*HR*) with 95% confidence intervals (CI). * *p* < 0.05. Additional analyses are provided in Supplementary Figure S3.

GO and KEGG analysis using the *R* programming language revealed that the six genes (*APOA4, SDPR, SERPINA1, EZR, SRGN and FGA*) are involved in many biological processes and pathways, as shown in clustering network graphs, including intestinal absorption, digestive system processes, digestion, postsynapse organization, peripheral nervous system axon regeneration, astral microtubule organization, regulation of intestinal lipid absorption, CoA-transferase activity, protein localization to cell, etc. The KEGG pathway analysis suggested that these genes may participate in disease-related pathways, including intestinal absorption, digestive system processes, postsynapse organization, peripheral nervous system axon regeneration, and digestion (Figure 6A,B). Next, we analyzed the expression of *SERPINA1, EZR* (Ezrin), *SRGN*, and *SDPR* in breast cancer and normal tissues, and found *SERPINA1* and *EZR* were highly expressed in breast cancer, while *SRGN* and *SDPR* were lowly expressed (Figure 6C). Analyses of the overall survival (OS) and disease-free survival (DFS) curves for *SERPINA1* revealed that high expression of *SERPINA1* was associated with a better prognosis (OS: log-rank *p* = 0.0015, DFS: log-rank *p* = 0.03) (Figure 6D). As shown in Figure 6E, SRGN expression was marginally significant for BC prognosis (*p* = 0.05, *HR* = 0.72, 95% CI: 0.52–1.00). The expression levels of *EZR* and *SDPR* demonstrated significant univariate associations with the log-rank test (EZR: *p* = 1.6 × 10^−5^, *HR* = 1.70, 95% CI: 1.22–2.37; SDPR: *p* = 0.02, *HR* = 0.64, 95% CI: 0.43–0.94). However, all survival analyses were univariate (log-rank test) without multivariate adjustment for clinical covariates; thus, these findings represent preliminary prognostic associations rather than independent prognostic indicators, and multivariate validation in independent cohorts is warranted.

## 4. Discussion

Breast cancer has emerged as the most common malignant tumor among women globally, with a notable trend towards a younger age at onset. Conventional diagnosis of breast cancer typically requires pathological examination of suspicious breast tissue specimens. However, the diagnostic performance of traditional tumor markers is insufficient to meet clinical demands and the criteria for early screening. The primary objective of our proteomic investigation into breast cancer is to enable rapid and accurate diagnosis of the disease while circumventing invasive procedures and to provide more efficient and accurate biomarkers [16].

With the rapid advancement of artificial intelligence, it has become feasible to conduct breast cancer diagnosis and biomarker screening based on serum proteomic data [38,39]. Circulating tumor DNA (ctDNA), transcriptomics, and other methods provide new ideas and approaches for the diagnosis and prognosis of breast cancer, but there are technical difficulties and high detection costs, which limit their clinical translation prospects. Artificial intelligence can leverage the high-throughput and high-sensitivity advantages of mass spectrometry-based proteomics to identify more discriminating diagnostic markers or data features from high-dimensional proteomic datasets, thereby enabling non-invasive diagnosis.

Previous studies have explored machine learning-based cancer diagnosis using proteomic data, but this work offers several distinct advantages [40,41]. For example, Senuri et al. developed a machine learning pipeline for ovarian cancer using public proteomics datasets [42]. Kim et al. generated a machine learning model predicting breast cancer (FN1, VWF, PRG4, MMP9, CLU, PRDX6, PPBP, APOC1 and CHL1) by mass spectrometry, based on 187 healthy controls and 215 breast cancer samples, showing an AUC of 0.9105 [43]. In contrast, this study integrated 9 machine learning algorithms to improve accuracy, employed multi-dimensional feature selection to enhance interpretability, and demonstrated model reliability through independent cohort validation, bridging the gap between basic research and clinical application. While the machine learning algorithms employed in this study are publicly available, the scientific novelty lies in the integrated translational pipeline. Rather than focusing on algorithmic innovation, this exploratory proof-of-concept case–control study highlights a biomarker panel with promising discriminative potential in a discovery setting, bridging serum proteomic profiles with tissue transcriptomic prognostic evidence in a hypothesis-generating manner.

There are many advantages to the combined machine learning-based breast cancer diagnostic model. Firstly, it offers high diagnostic accuracy and robustness. Nine machine learning algorithms were systematically compared during model development, covering both traditional and ensemble types, and achieved a total mean AUC of 0.90 ± 0.01, with ensemble algorithms such as Random Forest, LightGBM, and XGBoost reaching AUC values of over 0.97. This performance significantly outperforms the diagnostic efficacy of single traditional tumor markers. The favorable performance likely reflects the ability of the evaluated algorithms to capture complementary linear and nonlinear relationships in the proteomic feature matrix, overcoming the limitations of single algorithms in processing high-dimensional data [44]; Second, the stratified sampling and transcriptomic corroboration were used to assess model stability and reduce the risk of overfitting, reducing overfitting caused by data heterogeneity. The potential of machine learning algorithms lies in non-invasiveness and clinical accessibility. Unlike invasive diagnostic methods such as core needle biopsy [45], this model relies on serum samples, which are easy to collect and cause minimal patient discomfort. Serum collection can be completed in primary care settings without specialized equipment, and the MALDI-TOF MS analysis cost per sample ($25–40) is lower than MRI and PET-CT. This cost-effectiveness suggests potential utility in risk stratification settings, particularly in resource-limited regions where access to advanced imaging equipment is scarce. However, we emphasize that this potential can only be realized after prospective validation in real-world screening cohorts. Additionally, serum samples can be stored at −80 °C for up to 5 years, facilitating retrospective studies and multi-center data integration. Further more, it provides interpretable feature selection and biological relevance. By integrating model-based importance scores, SHAP values, and LIME values, this study identified differential peptide peaks and annotated them to 6 breast cancer-related genes (*APOA4, SDPR, SERPINA1, EZR, SRGN, FGA*). This multi-dimensional feature selection approach overcomes the “black box” limitation of traditional machine learning models. Moreover, MALDI-TOF MS is used for direct peptide profiling without antibody-dependent detection, eliminating cross-reactivity and antibody instability issues.

This study does not develop a novel machine learning algorithm or in the identification of previously unknown proteins, but rather in the establishment of a rigorous, multi-omics translational pipeline that integrates serum proteomic profiling, ML-based screening, physical peptide identification, tissue transcriptomic validation, and prognostic assessment within a single coherent framework. This closed-loop approach, combined with stringent analytical safeguards including nested cross-validation and independent temporal validation, provides a robust foundation for downstream clinical evaluation. While we acknowledge that immediate clinical utility under realistic screening conditions has not been demonstrated—and in particular, that the disease prevalence of our model decreases substantially from 80.3% in the case–control cohort to approximately 1.18–10.87% under true screening prevalence (0.1–1.0%)—we believe that the biomarker panel and the validated pipeline reported here represent a meaningful step toward the eventual translation of proteomics-based machine learning models into clinical practice. At present, this model is more appropriately considered as an auxiliary risk-stratification tool for high-risk populations rather than an independent screening or diagnostic assay.

Bioinformatics analysis further confirmed that these genes are involved in critical biological processes such as intestinal absorption, digestive system processes, and postsynaptic organization, and they participate in pathways closely related to postsynapse organization, peripheral nervous system axon regeneration, and digestion. For example, *SERPINA1* overexpression is associated with good prognosis and chemotherapy resistance in breast cancer [46], while *EZR* regulates tumor cell migration and invasion [47], linking diagnostic markers to disease mechanisms and enabling integrated diagnosis and treatment planning. The six proteins identified in this study—*APOA4*, *SDPR*, *SERPINA1*, *EZR*, *SRGN*, and *FGA*—encompass diverse biological pathways, including lipid metabolism, cytoskeletal linkage, protease inhibition, extracellular matrix remodeling, coagulation cascade, and caveolae regulation, reflecting the complex molecular heterogeneity underlying breast cancer pathogenesis [48]. Among them, *APOA4*, *EZR*, and *SRGN* are generally overexpressed in breast cancer and promote tumor proliferation, invasion, and chemoresistance through lipid metabolic reprogramming, JAK/STAT3 activation, and TGF-β-driven epithelial–mesenchymal transition (EMT), respectively [48,49,50]. Clinical studies have shown that high APOA4 expression is significantly associated with poorer cumulative overall survival in breast cancer patients (83.13% vs. 94.74%) [48]; high *EZR* expression correlates with shorter overall survival (*HR* = 1.21, *p* = 0.048) [50]; and *SRGN* overexpression is closely associated with poor prognosis in breast cancer patients receiving chemotherapy [51]. In contrast, SDPR acts as a metastasis suppressor and is frequently downregulated in breast cancer due to promoter methylation; its loss relieves the inhibition of TGF-*β* signaling, thereby facilitating EMT and distant metastasis [52]. Loss of *SDPR* expression correlates with significantly reduced distant-metastasis-free and relapse-free survival in breast cancer patients [52]. Notably, although *SERPINA1* is also overexpressed in tumor tissues, its elevated levels paradoxically correlate with better prognosis, suggesting a protective role possibly via inhibiting neutrophil elastase and mitigating inflammatory damage [46]. *SERPINA1* overexpression is associated with longer disease-free survival and overall survival [46]. Furthermore, specific cleavage fragments of *FGA* (encompassing residues 605–629) have been validated as serological markers for HER2-positive breast cancer, with their levels returning to normal after tumor resection, highlighting the dynamic interplay between the coagulation system and the tumor microenvironment [53]. These proteins do not function in isolation; rather, they form intricate regulatory networks through interconnected signaling cascades such as TGF-β, JAK/STAT3, AKT, integrin, and YAP/TEAD pathways, collectively shaping the malignant phenotype of breast cancer [49,50]. Thus, this panel of protein biomarkers not only provides a robust molecular foundation for the diagnostic model but also offers multidimensional insights into the pathophysiological dysregulation of metabolism, cytoskeletal dynamics, inflammation, and hemostasis in breast cancer, holding substantial potential for clinical translation.

This study has several limitations. The study recruited 255 breast cancer patients and 300 healthy controls from a single center (The Second Affiliated Hospital of Xi’an Jiaotong University), leading to potential demographic and clinical homogeneity biases. For instance, African American women have higher breast cancer mortality rates and distinct molecular profiles (e.g., higher prevalence of triple-negative breast cancer) [54], and the model’s efficacy in this group remains untested. Second, this proof-of-concept case–control biomarker study was not designed to discriminate benign (including benign conditions such as breast calcifications, acute mastitis, breast hyperplasia, and breast adenosis) from malignant lesions, nor to classify molecular subtypes. Breast cancer is a highly heterogeneous disease, with four main subtypes (Luminal A, Luminal B, HER2-positive, Triple-negative) differing in molecular characteristics and prognosis [55]. Third, technical limitations of mass spectrometry: Despite using MB-IMAC kits for peptide enrichment, MALDI-TOF MS still has limitations in detecting ultra-low-abundance peptides (<1 pg/mL), such as certain cytokines and growth factors, which may be valuable biomarkers [56]. Mass spectrometry performance is sensitive to experimental conditions (e.g., laser frequency, matrix preparation, instrument calibration), leading to potential variability between laboratories [57]. Fourth, the case–control design of the independent temporal validation cohort resulted in a markedly elevated prevalence (80.3%) compared to real-world breast cancer screening settings (0.1–1.0%). As a direct consequence, the PPV—which is prevalence-dependent—was substantially overestimated (80.3% in our cohort versus an estimated 1.18–10.87% under true screening prevalence). Therefore, the reported accuracy metrics derived from this cohort cannot be directly extrapolated to general population screening. This model is currently best suited as an auxiliary risk-stratification or rule-out tool, and its clinical utility in true screening scenarios requires prospective validation in a dedicated screening cohort. Fifth, we acknowledge that model calibration, prospective validation and batch-effect robustness were not formally evaluated. These limitations do not invalidate our findings but underscore the need for further validation before clinical translation. This lack of standardization may reduce the model’s reproducibility in multi-center validation, hindering its widespread clinical application. Nevertheless, we have established comprehensive SOPs to minimize such batch-to-batch or inter-laboratory variability. There was an absence of long-term prognostic and therapeutic response data. The model was evaluated solely for diagnostic accuracy, not for prognostic value or treatment response monitoring.

Actually, we recognize that the case–control design used in this discovery phase, while appropriate for initial biomarker identification, does not fully capture the diagnostic challenge encountered in clinical practice. Of note, we initially attempted a three-class classification model including benign conditions such as fibroadenoma, benign nodules, and calcifications. However, the benign group was not optimally matched to the other groups for key confounders, including age and menopausal status, both of which are known to affect serum proteomic profiles. Under these circumstances, a three-class comparison would have risked generating artifacts rather than robust biological signals. We therefore elected to prioritize a rigorously matched case–control design to establish a reliable biomarker panel as a necessary first step. A well-powered, carefully matched prospective study is currently planned as the logical next step, aiming primarily to establish and validate a standardized workflow that will serve as a foundation for future clinical translation.

To address these inherent limitations and translate our current proof-of-concept model into a clinically robust tool, we have defined a clear roadmap for subsequent investigations. First, we will initiate a multi-center prospective cohort study across diverse geographic and ethnic populations, with a particular focus on recruiting a screening-representative cohort with low disease prevalence to enable unbiased estimation of PPV and net clinical benefit. Second, upon achieving a sufficiently large sample size with adequate statistical power, we will perform subtype-stratified analyses, including benign breast lesions, to evaluate whether the diagnostic performance of our model differs across Luminal A, Luminal B, HER2-positive, and Triple-negative breast cancers. Ultimately, this stepwise strategy aims to develop a subtype-adaptive diagnostic model that can provide personalized decision support across the full spectrum of breast cancer heterogeneity. Future studies integrating these proteomic biomarkers with imaging (e.g., radiomics) or genomic (e.g., mutation or expression profiles) data may further enhance diagnostic performance through a multi-omics approach, which warrants systematic investigation in our subsequent work. In parallel, the six identified peptide biomarkers will be subjected to functional mechanistic investigations to unravel their biological contributions to breast cancer development and progression.

## 5. Conclusions

This study developed an AI-enhanced pipeline that integrates serum proteomics, multi-algorithm machine learning, and transcriptomic validation for the diagnosis and biological characterization of breast cancer. Our proteomics-driven diagnostic model, optimized by the LightGBM algorithm, identified a robust ten-peptide serum signature (APOA4, SDPR, SERPINA1, EZR, SRGN, and FGA) with high diagnostic accuracy (AUC = 0.97). The model maintained excellent performance (AUC = 0.88) in an independent external cohort, supporting its generalizability. Transcriptomic analyses further corroborated that these biomarkers are significantly dysregulated in tumor tissues and suggested potential prognostic relevance, linking them to key biological processes. In summary, this work provides a proof-of-concept demonstration that serum proteomic profiling, combined with machine learning, holds discriminative potential in a case–control discovery setting. However, given the substantially higher prevalence (80.3%) in the external cohort compared with real-world screening populations, the positive predictive value is expected to be considerably lower under low-prevalence conditions. Ongoing and future efforts are directed toward rigorous validation of the standardized workflow in screening cohorts that reflect true population prevalence, which is essential before any clinical translation can be considered.

## Figures and Tables

**Table 1:** Preliminary assignments of significantly different peptide peaks by LC-ESI-MS/MS sequencing.

*m*/*z* of the Top 10 Peaks	Gene
*m*/*z* 1515.03	——
*m*/*z* 4981.77	*APOA4*
*m*/*z* 1630.67	*FGA*
*m*/*z* 1548.26	*SERPINA1*
*m*/*z* 1633.42	——
*m*/*z* 1871.21	*FGA*
*m*/*z* 1946.75	*EZR*
*m*/*z* 2961.89	*SRGN*
*m*/*z* 3008.39	*SDPR*
*m*/*z* 4110.83	——

NOTE: ——, not detected.

## Data Availability

The datasets generated and/or analyzed during this study are available from the corresponding author upon reasonable request.

## Supplementary Materials

The following supporting information can be downloaded at: https://www.mdpi.com/article/10.3390/bioengineering13091040/s1, Figure S1: Participant flow diagram for analysis; Figure S2: Calibration and Bootstrap-Based Feature-Stability Analyses; Figure S3: Differential expression analysis of FGA and APOA4 between breast cancer tissues (from TCGA-BRCA) and normal breast tissues (from GTEx); Table S1: Model-specific hyperparameter search spaces; Table S2: Baseline for external validation cohort; Table S3: Threshold-dependent nested-CV performance; Table S4: Nested cross-validation discrimination; Table S5: Phisiology of the found protein; Supplementary File S1: LOO+forward analyses; Supplementary File S2: LC-ESI-MS.

## Abbreviations

The following abbreviations are used in this manuscript:

AI	Artificial Intelligence
ASCO	American society of clinical oncology
BC	Breast cancer
CEA	Carcinoembryonic antigen
DFS	Disease-free survival
E2	Estradiol 2
EGFR	Epidermal growth factor receptor
ER	Estrogen receptor
HER2	Human epidermal growth factor receptor
OS	Overall survival
PET/CT	Positron emission tomography/computed tomography
PR	Progesterone receptor
TNBC	Triple-negative BC
TCGA	The Cancer Genome Atlas
KEGG	Kyoto Encyclopedia of Genes and Genomes
GO	Gene Ontology
ML	Machine Learning
AUC	Area Under The Curve
MRI	Magnetic Resonance Imaging
DIA	Data-Independent Acquisition
DDA	Data-Dependent Acquisition
MRM	Multiple Reaction Monitoring
PRM	Parallel Reaction Monitoring
FDR	False Discovery Rate
PCA	Principal Component Analysis
DCA	Decision curve analysis
K-M	Kaplan–Meier
