# Supplementary material for: From Machine Learning-Enhanced Proteomics to a Validated Diagnostic Model: A Pipeline for Breast Cancer Biomarker Discovery via Independent and Transcriptomic Corroboration
Source: Bioengineering (Basel). 2026 Sep 7;13(9):1040. doi: 10.3390/bioengineering13091040 (PMC13603888; doi:10.3390/bioengineering13091040)
Supplement: Supplementary file 1 [file bioengineering-13-01040-s001.zip › Supplementary Figures S1-S3..pdf]

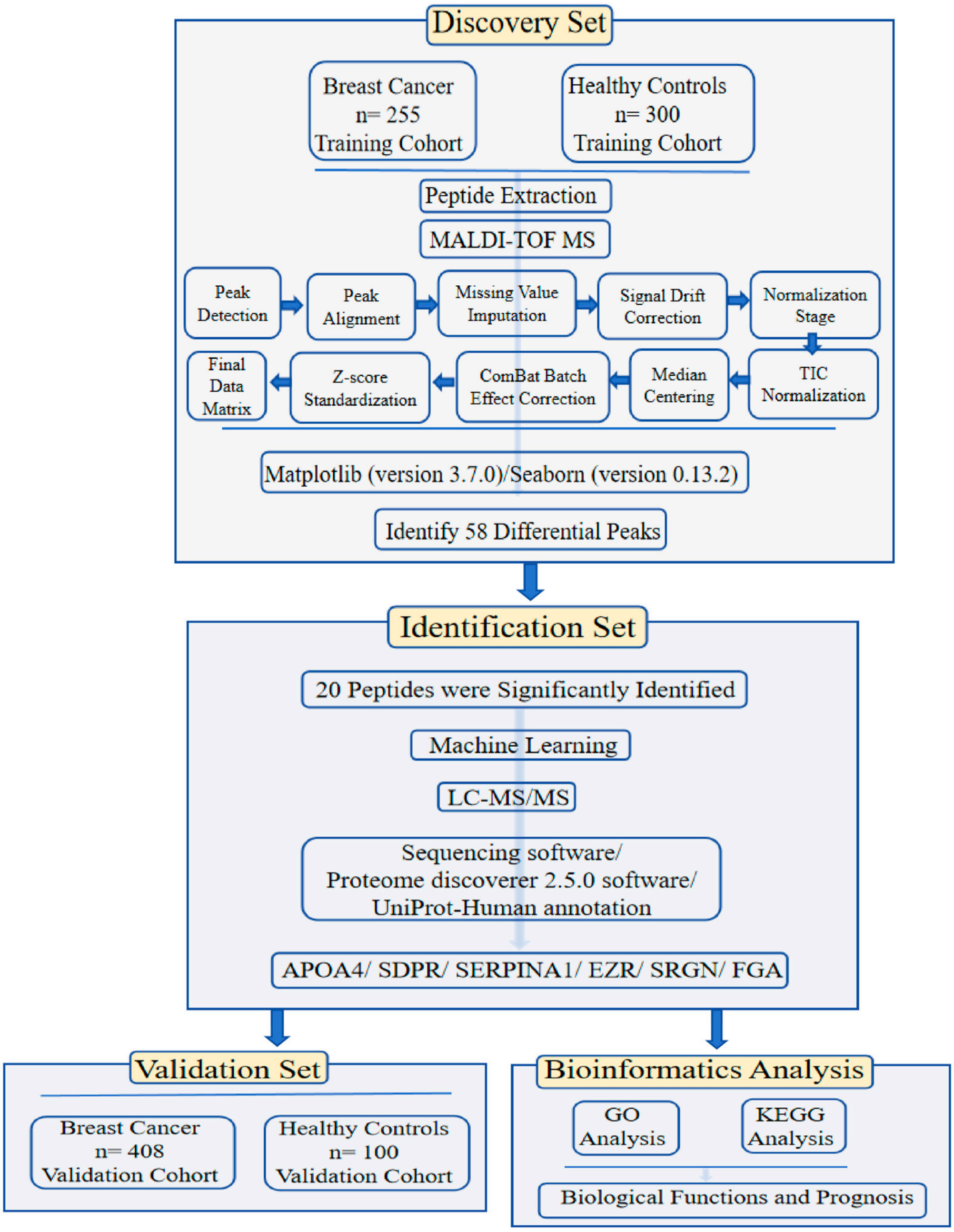

Supplementary Figure S1. Participant flow diagram for analysis.

Note: Workflow used for the discovery, identification, validation and bioinformatic analysis of potential biomarkers for breast cancer.

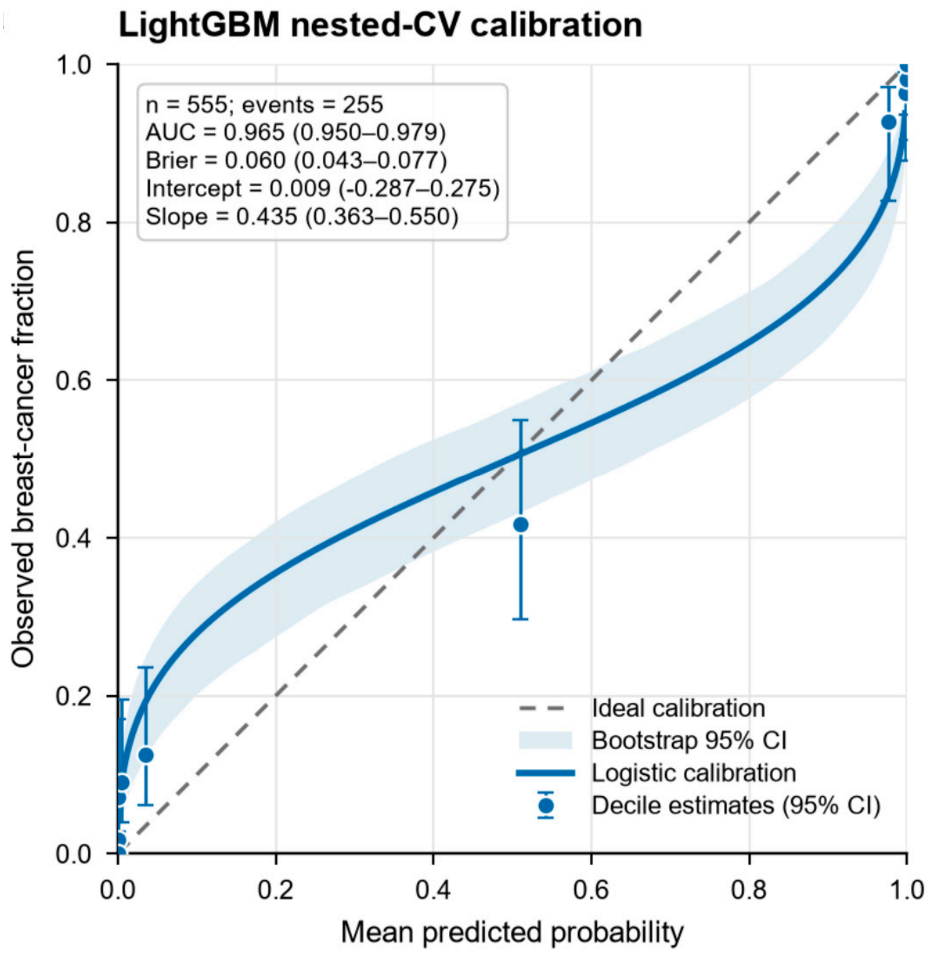

Supplementary Figure S2. Calibration and Bootstrap-Based Feature-Stability Analyses.

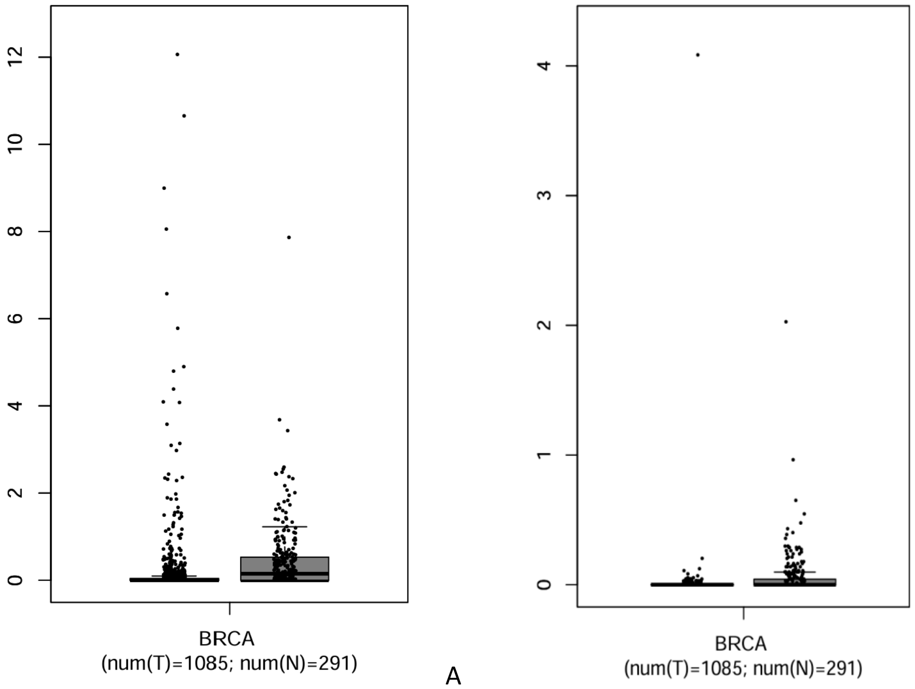

A B

Supplementary Figure S3. Differential expression analysis of FGA and APOA4 between breast cancer tissues (from TCGA-BRCA) and normal breast tissues (from GTEx). (A) Expression analysis of FGA between breast cancer tissues and normal breast tissues. (B) Expression analysis of APOA4 between breast cancer tissues and normal breast tissues.
