# Supplementary material for: From Machine Learning-Enhanced Proteomics to a Validated Diagnostic Model: A Pipeline for Breast Cancer Biomarker Discovery via Independent and Transcriptomic Corroboration
Source: Bioengineering (Basel). 2026 Sep 7;13(9):1040. doi: 10.3390/bioengineering13091040 (PMC13603888; doi:10.3390/bioengineering13091040)
Supplement: Supplementary file 1 [file bioengineering-13-01040-s001.zip › Supplementary File S1-LOO+forward analyses.pdf]

The 10-peak panel used in the primary manuscript was defined by the original composite feature-selection workflow integrating SHAP, LIME, and decision-curve analysis. The reviewer-requested perturbation analysis was conducted after completion of the primary analysis and was treated as a supplementary robustness analysis. No primary model performance estimate or main figure was recomputed or replaced.

The discovery dataset comprised 555 samples (255 malignant and 300 healthy controls). The current panel contained ten m/z features: 4110.76, 1633.42, 1515.03, 4981.93, 1630.11, 1547.58, 1871.23, 1946.12, 2961.68, and 3008.09. Eleven remaining candidate features were individually considered for addition: m/z 5354.80, 2083.62, 2661.80, 1875.43, 1517.14, 5921.55, 1950.24, 1568.74, 2585.32, 6686.58, and 6717.04.

For the exhaustive fixed-panel comparison, we evaluated the current 10-peak panel, ten leave-one-peak-out panels, and eleven add-one-candidate panels. Each panel was assessed using repeated stratified five-fold cross-validation with 10 repeats and a common random seed. The same fold assignments were used for every panel. Missing values, if present, were imputed with the median calculated in the training fold. Standardization parameters were also estimated only from the training fold. LightGBM hyperparameters were locked at learning_rate = 0.1, min_child_samples = 10, n_estimators = 200, num_leaves = 31, and random_state = 42; no additional hyperparameter search was performed during panel perturbation.

For nested validation, the data were divided into five stratified outer folds. In each outer iteration, four-fold inner cross-validation was used to select (i) the best 9-peak panel among the ten leave-one-out alternatives and (ii) the best 11-peak panel among the eleven add-one alternatives. Selection was based only on the mean inner-fold ROC-AUC. Each selected panel and the unchanged 10-peak panel were then trained on the complete outer training partition and evaluated in the held-out outer partition. Predictions from all outer folds were pooled to obtain one out-of-fold prediction per patient.

ROC-AUC was the primary metric. Accuracy, precision, sensitivity, specificity, F1 score, average precision, and Brier score were calculated using a probability threshold of 0.5 where applicable.

**Supplementary Results**

In repeated cross-validation of all fixed perturbations, the current 10-peak panel achieved an averaged out-of-fold ROC-AUC of 0.980. Removing m/z 1633.42 caused the largest deterioration (AUC =0.968; paired delta AUC -0.0115, 95% CI: -0.0189 to -0.0051), whereas no leave-one-out panel improved on the baseline. The highest fixed add-one performance was obtained by adding m/z 6717.04 (AUC 0.990; paired delta AUC +0.0109, 95% *CI*: +0.0045 to 0.0185), followed by m/z 6686.58 (AUC 0.989; paired delta AUC +0.0093, 95% *CI:* +0.0043 to +0.0156).

In the more conservative nested analysis, the 10-peak baseline achieved an AUC of 0.976 (95% CI 0.962-0.987), accuracy of 0.937, sensitivity of 0.910, specificity of 0.960, and F1 score of 0.930. The nested-selected 9-peak panel yielded an AUC of 0.975 (0.961-0.986), with a paired delta AUC of -0.0015 (-0.0053 to 0.0020). The feature selected for removal differed in every outer fold, providing no evidence for a single stably redundant feature. The nested-selected 11-peak panel yielded an AUC of 0.988 (0.979-0.995), accuracy of 0.951, sensitivity of 0.941, specificity of 0.960, and F1 score of 0.947. Its paired AUC improvement was +0.0119 (0.0064-0.0185). Among the added candidates, m/z 6686.58 was selected in three of five outer folds, while m/z 6717.04 and 1875.43 were each selected once.

| **Panel** | **AUC (95% CI)** | **Delta AUC vs 10 peaks (95% CI)** | **Accuracy** | **Sensitivity** | **Specificity** | **F1 score** |
|---|---|---|---|---|---|---|
| Nested-selected 9 peaks | 0.975 (0.961-0.986) | -0.0015 (-0.0053 to 0.0020) | 0.919 | 0.898 | 0.937 | 0.911 |
| Current 10 peaks | 0.976 (0.962-0.987) | Reference | 0.937 | 0.910 | 0.960 | 0.930 |
| Nested-selected 11 peaks | 0.988 (0.979-0.995) | +0.0119 (0.0064-0.0185) | 0.951 | 0.941 | 0.960 | 0.947 |

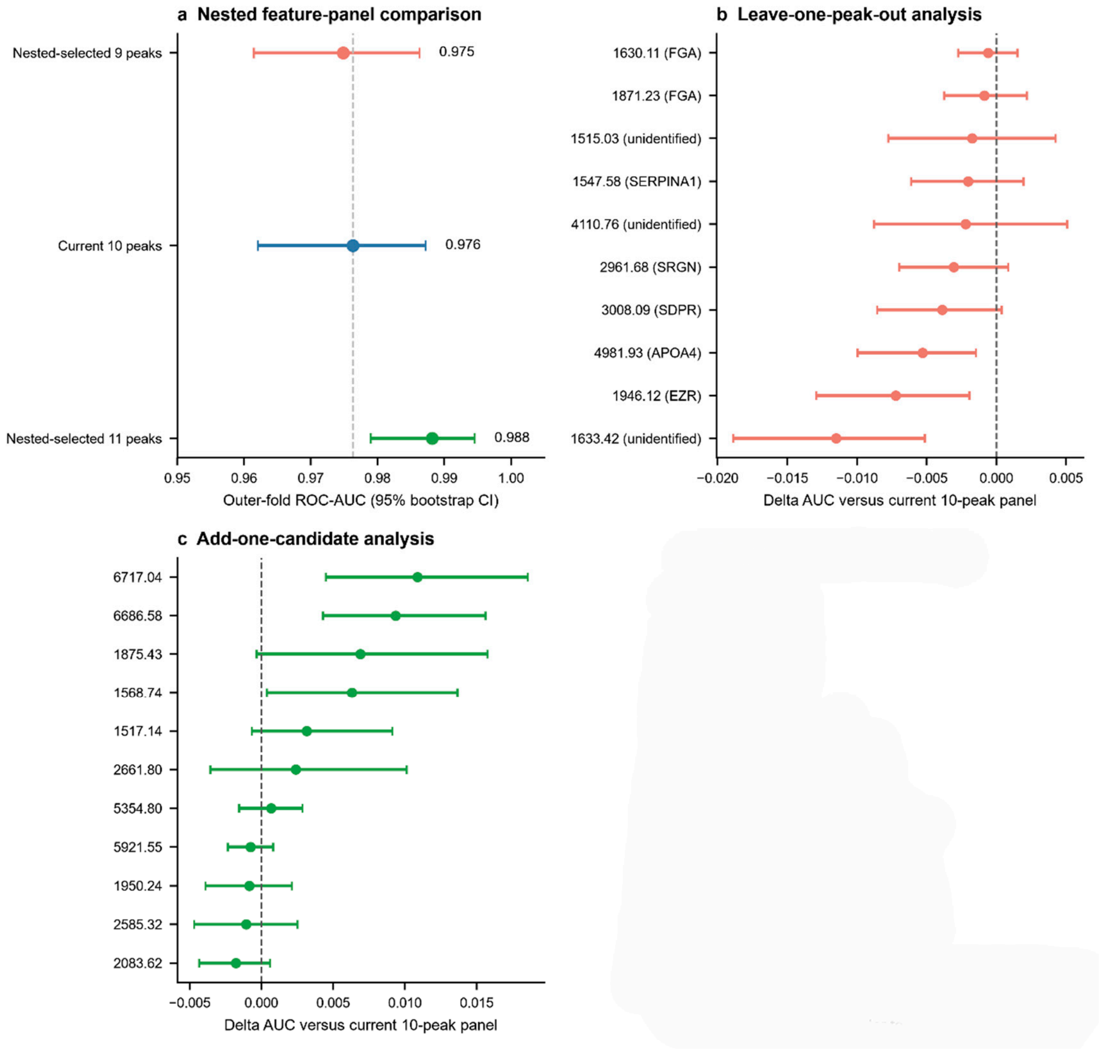


**Robustness of the prespecified 10-peak diagnostic panel to feature removal and addition.** (a) Outer-fold ROC-AUC values and 95% patient-level bootstrap confidence intervals for the nested-selected 9-peak panel, the unchanged 10-peak panel, and the nested-selected 11-peak panel. (b) Paired change in ROC-AUC for each leave-one-peak-out panel relative to the current 10-peak panel in repeated stratified five-fold cross-validation. (c) Paired change in ROC-AUC for each add-one-candidate panel.
