# Supplementary material for: From Machine Learning-Enhanced Proteomics to a Validated Diagnostic Model: A Pipeline for Breast Cancer Biomarker Discovery via Independent and Transcriptomic Corroboration
Source: Bioengineering (Basel). 2026 Sep 7;13(9):1040. doi: 10.3390/bioengineering13091040 (PMC13603888; doi:10.3390/bioengineering13091040)
Supplement: Supplementary file 1 [file bioengineering-13-01040-s001.zip › Supplementary File S2-LC-ESI-MS.pdf]

Peptide Identification Project

1.Instrument And Equipment

1. Equipment

Instrument	Model	Manufacturer
High-speed centrifuge	Pico 17 Centrifuge	Thermo Scientific
Vortex mixer	GENIE	GENIE
Pipettes	2.5, 10, 20, 200, 1000 µL	Eppendorf
Electronic balance	SQP	Sartorius Scientific Instruments
Heat concentrator	SC210A	Thermo Scientific
pH meter	Orion Star LogR pH Benchtop	Thermo Scientific
NanoDrop	2000C	Thermo Scientific

2. Consumables

Consumable	Specification	Manufacturer
Disposable tips	0.5–10 µL, 2–200 µL, 100–1000 µL	Axygen
30 kDa ultrafiltration	Sartorius	VN01H22
C18 Cartridge	WAT023590	Waters
Centrifuge tubes	1.5 mL	Eppendorf
Vials	—	Thermo Scientific

2. Data Analysis

Raw MS Data

The raw MS data are in .RAW format. Database searching was performed using Sequest and Proteome Discoverer 2.5.0 (Thermo Scientific).

Database

Database used: UniProt protein database for the corresponding species (downloaded) [uniprotkb_UP000005640_9606.fasta]

Search Parameters

Raw files were submitted via Proteome Discoverer to the Sequest server, against the selected database. Parameters are listed in Table 2-2.

3. Search parameters

Item	Value
**Enzyme**	No Enzyme
**Variable modifications**	Oxidation (M); Acetyl (Protein N-term)，Deamidated（N）

**Peptide Mass Tolerance**	± 15ppm
**Fragment Mass Tolerance**	0.02 Da
**peptide confidence**	high
**peptide length**	>4

Result filtering: Peptide FDR ≤ 0.01.

3. Peptide Identification Results

Molecular weight	Sequence	Position	Protein ID	Protein name
1548.26	K.SPLFMGKVVNPTQK.	405-418	P01009	SERPINA1
1630.67	EADHEGTHSTKRGHA	610-624	P02671	FGA
1871.21	KMADEAGSEADHEGTHST	602-609	P02671	FGA
1946.75	K.IAQDLEMYGINYFEIK.N	194-209	P15311	EZR Ezrin
2961.89	HDNLRSLDRNLPSDSQDLGQHGLEED	130-155	P10124	SRGN
3008.39	SYALTSEEAERSDGDPVQPAVLQVHQTS	398-425	O95810	SDPR
4981.77	FSTFKEKESQDKTLSLPELEQQQEQQQEQQQEQVQMLAPLES.	355-396	P06727	APOA4

Note: Protein functions can be viewed in the UniProt database using the 'Protein ID' at https://www.uniprot.org/.
