# Supplementary material for: From Machine Learning-Enhanced Proteomics to a Validated Diagnostic Model: A Pipeline for Breast Cancer Biomarker Discovery via Independent and Transcriptomic Corroboration
Source: Bioengineering (Basel). 2026 Sep 7;13(9):1040. doi: 10.3390/bioengineering13091040 (PMC13603888; doi:10.3390/bioengineering13091040)
Supplement: Supplementary file 1 [file bioengineering-13-01040-s001.zip › Supplementary Tables S1 and S3-S5.pdf]

Supplementary Table S1. Model-specific hyperparameter search spaces

Algorithm	Candidate values
Logistic regression	C: 0.1, 1, 10; class weight: none, balanced
Random Forest	n_estimators: 100, 300; max_depth: none, 8; min_samples_leaf: 1, 3
Decision Tree	max_depth: 3, 8, none; min_samples_leaf: 1, 5
SVM	C: 0.1, 1, 10; kernel: linear, RBF; gamma: scale
KNN	n_neighbors: 3, 5, 9; weights: uniform, distance
GaussianNB(GNB)	var_smoothing: 1×10^{-11}, 1×10^{-9}, 1×10^{-7}
AdaBoost	n_estimators: 50, 150; learning_rate: 0.05, 0.5, 1.0
XGBoost	n_estimators: 100, 200; max_depth: 2, 4; learning_rate: 0.03, 0.10; subsample: 0.8
LightGBM(LGBM)	n_estimators: 100, 200; num_leaves: 7, 31; learning_rate: 0.03, 0.10; min_child_samples: 10, 20

Supplementary Table S1 footnotes: Algorithm-specific grids were used because identical hyperparameters are not applicable across different classifier families. All candidate models were evaluated using the same nested data partitions and ROC-AUC optimization criterion. Abbreviations: Gaussian Naive Bayes; LGBM, Light Gradient Boosting Machine; SVM, Support Vector Machine.

Supplementary Table S3. Threshold-dependent nested-CV performance.

**Algorithm**	**Accuracy**	**Precision**	**Sensitivity**	**Specificity**	**F1**
XGBoost	0.932	0.936	0.914	0.947	0.925
Random Forest	0.941	0.948	0.922	0.957	0.934
LightGBM	0.930	0.935	0.910	0.947	0.922
SVM	0.915	0.913	0.902	0.927	0.907
AdaBoost	0.917	0.913	0.906	0.927	0.909
KNN	0.879	0.905	0.824	0.927	0.862
Logistic regression	0.881	0.899	0.835	0.920	0.866
Decision Tree	0.861	0.845	0.855	0.867	0.850
GaussianNB	0.800	0.867	0.667	0.913	0.754

Supplementary Table S4. Nested cross-validation discrimination.

Algorithm	Out-of-fold AUC	Bootstrap 95% CI
XGBoost	0.974	0.961-0.986
Random Forest	0.974	0.959-0.985
LightGBM	0.965	0.950-0.979
SVM	0.965	0.949-0.979
AdaBoost	0.955	0.934-0.973
KNN	0.944	0.924-0.963
Logistic regression	0.919	0.890-0.943
Decision Tree	0.904	0.875-0.929
GaussianNB	0.875	0.844-0.902

AUC values were calculated from pooled outer-fold predictions. Confidence intervals were obtained from 500 bootstrap resamples.

Supplemently Table S5. Phisiology of the found protein.

Protein	Functional Category	Expression in Breast Cancer	Prognostic Significance	Key Molecular Mechanism
APOA4	Lipid metabolism	Overexpressed	Poor prognosis	Lipid metabolic reprogramming
SDPR	Metastasis suppressor / Caveolae formation	Downregulated	Favorable (when high)	Inhibition of TGF-β/EMT signaling
SERPINA1	Protease inhibition / Acute-phase response	Overexpressed	Favorable prognosis	Anti-protease and anti-inflammatory effects
EZR	Cytoskeletal linker (ERM family)	Overexpressed	Poor prognosis	Activation of JAK/STAT3 and AKT pathways
SRGN	Proteoglycan / ECM remodeling	Overexpressed	Poor prognosis	TGF-β/EMT induction, chemoresistance via YAP/TEAD
FGA	Coagulation / Fibrinogen subunit	Altered fragment levels	Biomarker for HER2+	Integrin-AKT signaling

Apolipoprotein A-IV (APOA4), Serum Deprivation Response Protein (SDPR), Alpha-1-Antitrypsin (SERPINA1), Ezrin (EZR), Serglycin (SRGN), and Fibrinogen Alpha Chain (FGA)
